# Prognostic significance of the systemic immune inflammation index in patients with metastatic and unresectable pancreatic cancer

**DOI:** 10.3389/fsurg.2022.915599

**Published:** 2022-08-30

**Authors:** Rongshuang Han, Zibin Tian, Yueping Jiang, Ge Guan, Xiaowei Wang, Xueguo Sun, Yanan Yu, Xue Jing

**Affiliations:** ^1^ Gastroenterology Department, The Affiliated Hospital of Qingdao University, Qingdao, China; ^2^ Liver Disease Center Department, The Affiliated Hospital of Qingdao University, Qingdao, China

**Keywords:** pancreatic cancer, inflammatory markers, systemic immune inflammation index, survival, metastasis

## Abstract

**Purpose:**

Systemic inflammatory markers may be predictors of the survival rate of patients with pancreatic cancer (PC). The aim of this work was to investigate the prognostic value of markers, mainly the systemic immune inflammation index (SII), in patients with metastatic and unresectable PC and to explore the relationship between markers and liver metastasis.

**Methods:**

Records of patients with metastatic and unresectable PC at the Affiliated Hospital of Qingdao University from January 2000 to December 2019 and who were followed until December 2020 were retrospectively analyzed. Clinical data and laboratory indexes were collected, and cut-off values for inflammatory markers were determined using median values. The Cox proportional hazard model was used to analyze the prognostic value of the markers through univariate and multivariate survival analysis.

**Results:**

All 253 patients met the inclusion criteria, and 102 (42.0%) patients had liver metastasis. The patients were divided into a high SII group and a low SII group, and the cut-off value was 533. In the multivariate analysis, high SII (HR = 2.151; *p *< 0.001), chemotherapy (HR = 0.546; *p *< 0.001), lymph node metastasis (HR = 4.053; *p *< 0.001), and distant metastasis (HR = 1.725; *p *= 0.001) were independent risk markers of overall survival (OS). The level of markers, mainly SII, PLR and NLR, were higher in patients with liver metastasis.

**Conclusions:**

A high level of SII is an independent risk factor for short overall survival of patients with metastatic and unresectable PC. Patients with a high level of the inflammatory markers SII, PLR, and NLR, may be more prone to early liver metastasis.

## Introduction

Pancreatic cancer (PC), especially pancreatic adenocarcinoma, has a poor prognosis. It is the fourth leading cause of cancer death in the western world (1), and the prediction curve of relevant reports indicates that it will become the second most common cause after lung cancer around 2030 (2). According to the latest guidelines for the treatment of PC in China, systematic treatment, including surgical resection, systemic chemotherapy, and radiation therapy, is the whole-course management model for most patients (3, 4). However, the five-year survival rate of PC is extremely low, at less than 6% (5), and the median survival for patients with metastatic diseases is only 6–12 months (6). PC is categorized as resectable, borderline resectable, locally advanced, or metastatic (7). There is no reliable predictor of prognosis for patients with metastatic and unresectable PC. Therefore, prognostic factors accompanying PC play a crucial role in guiding treatment.

Chronic inflammation has been linked to various steps in tumorigenesis, including cellular transformation, promotion, survival, proliferation, invasion, angiogenesis, and metastasis (8, 9). The systemic immune inflammation index (SII), based on the counts of platelets, neutrophils, and lymphocytes, has been linked to the prognosis of patients with cancers, such as lung cancer, hepatocellular carcinoma, colorectal cancer, and esophageal cancer (10–13). In PC, some studies have reported that SII is associated with poor prognosis (14–17). The prognostic value of the neutrophil-to-lymphocyte count (NLR), the platelet-to-lymphocyte count (PLR), the lymphocyte-to-monocyte ratio (LMR), and the prognostic nutritional index (PNI) for prognosis is controversial (18–21). Therefore, in this study, we compared systemic inflammatory markers and validated their prognostic significance in PC patients with metastatic and unresectable disease.

## Methods

### Patient selection

This was a retrospective study of patients seen at the Affiliated Hospital of Qingdao University from January 2000 to December 2019 who had PC that was metastatic or unresectable, including palliative surgical resection and non-surgical treatment. The diagnosis of PC was confirmed by histopathologic examination. Exclusion criteria were (1) follow up not possible, for example, by loss of contact; (2) PC combined with other malignant tumors; and (3) PC complicated by hematological diseases. The researchers contacted the patients *via* telephone and a short messaging service. This study was reviewed and approved by the Institutional Review Board of the Affiliated Hospital of Qingdao University. Data obtained from the medical records included clinical characteristics, histopathological findings, administration of adjuvant chemotherapy, and clinical outcomes.

### Data collection

The clinical data of patients with PC at admission were collected, including demographic data, staging (TNM) according to the 8th edition of the Union for International Cancer Control /American Joint Committee on Cancer, history of smoking and drinking, concomitant illnesses, whole blood cell count, tumor location, tumor size, CA19-9 level, total bilirubin, alanine aminotransferase (ALT), aspartate aminotransferase (AST), and albumin (Alb). All laboratory tests used in this analysis were recorded in routine examinations before the diagnosis of pancreatic cancer was made. SII was calculated as the ratio of platelet × neutrophil/lymphocyte count. NLR was calculated as the ratio of neutrophil/lymphocyte count. PLR was calculated as the ratio of platelet/lymphocyte count. LMR was calculated as the ratio of lymphocyte/monocyte count. PNI was calculated as the serum albumin concentration (g/L) + 5 × lymphocyte count (109/L). The main outcome was overall survival (OS), defined as the time between diagnosis and final follow-up of PC.

### Statistical analyses

Optimal cutoff values for SII, NLR, PLR, LMR, and PNI were determined using median values. Univariate analysis of clinical baseline data was carried out with t tests for continuous data with normally distributed values and chi-square tests for categorical data in SII groups. Univariate and multivariate analyses were performed with the Cox proportional-hazards regression model. Cumulative survival rates were calculated with the Kaplan–Meier method, and between-group differences were assessed with the log-rank test. After univariate analysis was performed, meaningful variables were included in the multivariate analysis. Giving the inflammatory markers are significantly associated with each other, we used covariance analysis before multivariate analysis. Death from causes other than PC and survival until the end of the observation period were considered censored observations. Cox proportional-hazards regression was used to determine the prognostic factors associated with OS with univariate and multivariate analyses. The hazard ratio (HR) and 95% confidence interval (CI) were used to describe the relative risk factors. All tests were two-sided, and statistical significance was inferred at a *p* value of <0.05. SPSS (24.0) was used to analyze data.

## Results

### Clinicopathological characteristics

A total of 243 patients were enrolled. The average age of the patients was 61.7 (SD 10.6) years; 158 (62.6%) were male. Patients with yellow skin or sclera were identified as having jaundice. Based on median values, the cutoff values were 533 (SII), 2.63 (NLR), 138 (PLR), 3.33 (LMR), and 46.4 (PNI). The patients were divided into high and low SII groups using 533 as a cut-off value; 120 patients were in the high SII group. Demographic and clinical characteristics of the enrolled patients are listed in Table 1.

**Table 1:** Baseline clinicopathological characteristics.

Factors	All	SII		*p*
		Low	High	
*n*	243	123	120	
Age, mean (±SD)	61.74 (10.6)			0.471
Sex, *n* (%)				0.804
Male	152 (62.6%)	76	76	
Female	91 (37.4%)	47	44	
Jaundice, *n* (%)				0.158
Yes	67 (27.6%)	29	38	
No	176 (72.4%)	94	82	
CA19-9, U/ml	187			0.229
≤114	56 (23.0%)	34	22	
>114	131 (53.9%)	67	64	
Alb, g/L	241			0.016
≥30	229 (94.2%)	120	109	
<30	12 (4.9%)	2	10	
TBIL, μmol/L				0.150
0–35	169 (69.5%)	92	77	
36–200	42 (17.3%)	16	26	
>200	32 (13.2%)	15	17	
ALT, U/L				0.684
0–100	181 (74.5%)	93	88	
>100	62 (25.5%)	30	32	
AST, U/L				0.795
0–80	182 (74.5%)	93	89	
>80	62 (25.5%)	30	31	
Pancreatitis				0.262
Yes	25 (10.3%)	10	15	
No	218 (89.7%)	113	105	
Diabetes				0.851
Yes	60 (24.7%)	31	29	
No	183 (75.3%)	92	91	
Hypertension				0.197
Yes	62 (25.5%)	27	35	
No	181 (74.5%)	96	85	
Smoke				0.153
Yes	71 (29.2%)	41	30	
No	172 (70.8%)	82	90	
Drink				0.621
Yes	58 (23.9%)	31	27	
No	185 (76.1%)	92	93	
Tumor location				0.047
Head	171 (70.4%)	95	76	
Body	40 (16.5%)	17	23	
Tail	32 (13.2%)	11	21	
Tumor size				0.101
≤2 cm	2 (0.08%)	1	1	
≤4 cm and >2 cm	34 (14.0%)	23	11	
>4 cm	207 (85.2%)	99	108	
T				0.114
1	4 (1.6%)	2	2	
2	74 (30.5%)	41	33	
3	91 (37.4%)	37	54	
4	74 (30.5%)	43	31	
N				0.106
0	33 (13.6%)	22	11	
1	179 (73.7%)	88	91	
2	31 (12.8%)	13	18	
M				0.017
0	89 (36.6%)	54	35	
1	154 (63.4%)	69	85	
Chemotherapy	155			0.005
Yes	102 (42.0%)	63	39	
No	100 (41.2%)	42	58	
Radiotherapy				0.893
Yes	21 (8.6%)	11	10	
No	181 (74.5%)	92	89	
Liver metastasis				0.003
Yes	102 (42.0%)	40	62	
No	140 (57.6%)	82	58	

ALT, alanine transaminase; AST, aspartate transaminase; SII, the systemic immune-inflammation index; TBIL, total bilirubin; Alb, albumin.

There were significant differences in Alb (*p *= 0.016) and tumor location (*p *= 0.047) between the high SII group and the low SII group. In addition, according to the Union for International Cancer Control criteria, distant metastasis was significantly (*p *= 0.017) more common in the high SII group (Table 1).

### Systemic inflammatory markers are associated with OS

As shown in Table 2, univariate analysis identified high SII (HR = 2.448; *p *< 0.001), high PLR (HR = 1.912; *p *< 0.001), high NLR (HR = 1.759; *p *< 0.001), high LMR (HR = 0.735; *p *= 0.018), chemotherapy (HR = 1.580; *p *= 0.002) and radiotherapy (HR = 2.029; *p *= 0.003) as factors affecting OS. Moreover, lymph node metastasis (LNM) (HR = 3.232 *p *< 0.001) and distant metastasis (HR = 1.975; *p *< 0.001) were also factors affecting the OS. Before multivariate analysis, we used covariance analysis to analyze the relationship between indicators and OS to exclude the impact of the interaction between markers on the final results. The results showed that except for SII, the *p* values of other indicators were >0.05. So we include these meaningful indicators into the multivariate analysis.The results showed that high SII (HR = 2.151; *p *< 0.001), chemotherapy (HR = 0.546; *p *< 0.001), LNM (HR = 4.053; *p *< 0.001) and distant metastasis (HR = 1.725; *p *= 0.001) were independent risk markers of OS.

**Table 2:** Univariate and multivariate cox proportional-hazard regression analysis of OS.

	Univariate analysis, HR (95% CI)	*p*	Multivariate analysis, HR (95% CI)	*p*
Sex	1.093 (0.841–1.420)	0.506	–	
Jaundice				
Yes vs. no	0.913 (0.685–1.215)	0.531	–	
Alb, g/L				
≥30 vs. <30	1,292 (0.722–2.313)	0.388	–	
TBIL, µmol/L				
0–35 vs. 36–200	0.927 (0.654–1.315)	0.671	–	
0–35 vs. >200	1.035 (0.709–1.512)	0.858	–	
ALT, U/L				
0–100 vs. >100	0.883 (0.656–1.189)	0.413	–	
AST, U/L				
0–80 vs. >80	0.937 (0.697–1.259)	0.665	–	
Tumor location				
Head vs. Body	1.355 (0.954–1.923)	0.089		
Head vs. Tail	1.192 (0.815–1.741)	0.365		
Tumor size				
≤2 cm vs. ≤4 cm and >2 cm	1.206 (0.288–5.051)	0.798	–	
≤2 cm vs. >4 cm	1.497 (0.371–6.035)	0.571	–	
Diabetes, yes vs. no	1.058 (0.789–1.418)	0.708	–	
SII, ≤533 vs. >533	2.448 (1.878–3.189)	0.000	2.151 (1.592–2.906)	0.000
PLR, ≤138 vs. >138	1.912 (1.476–2.476)	0.000	1.105(0.781–1.563)	0.581
NLR, ≤2.63 vs. >2.63	1.759 (1.362–2.272)	0.000	1.316(0.881–1.966)	0.241
PNI, ≤46.41 vs. >46.41	0.782 (0.607–1.007)	0.057	–	
LMR, ≤3.33 vs. >3.33	0.735 (0.570–0.948)	0.018	1.176 (0.822–1.683)	0.946
Chemotherapy				
Yes vs. no	0.463 (0.346–0.620)	0.000	0.546 (0.400–0.745)	0.000
Radiotherapy				
Yes vs. no	0.614 (0.389–0.968)	0.036	0.754 (0.458–1.239)	0.283
Liver metastasis				
Yes vs. no	1.714 (1.315–2.235)	0.000	0.981 (0.667–1.442)	0.913
T				
1 vs. 2	0.759 (0.277–3.083)	0.593	–	
1 vs. 3	0.938 (0.343–2.563)	0.901		
1 vs. 4	1.233 (0.450–3.380)	0.684		
N				
0 vs. 1	3.232 (2.094–4.989)	0.000	3.215 (1.937–5.336)	0.000
0 vs. 2	5.698 (3.248–9.995)	0.000	4.053 (2.094–7.844)	0.000
M				
0 vs. 1	1.975 (1.497–2.605)	0.000	1.725 (1.239–2.400)	0.001

ALT, alanine transaminase; AST, aspartate transaminase; SII, the systemic immune-inflammation index; TBIL, total bilirubin; Alb, albumin; NLR, neutrophil/lymphocyte count; PLR, platelet/lymphocyte count; LMR, lymphocyte/monocyte; PNI, albumin (g/L) + 5 × lymphocyte count (10^9^/L).

**Figure 1:**
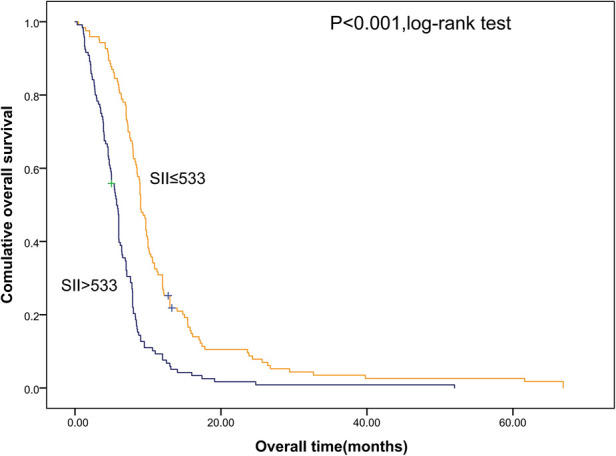
Kaplan–Meier graphs of OS for patients with high SII (>533) and low SII (≤533). OS, overall survival; SII, systemic immune-inflammation index.

**Figure 2:**
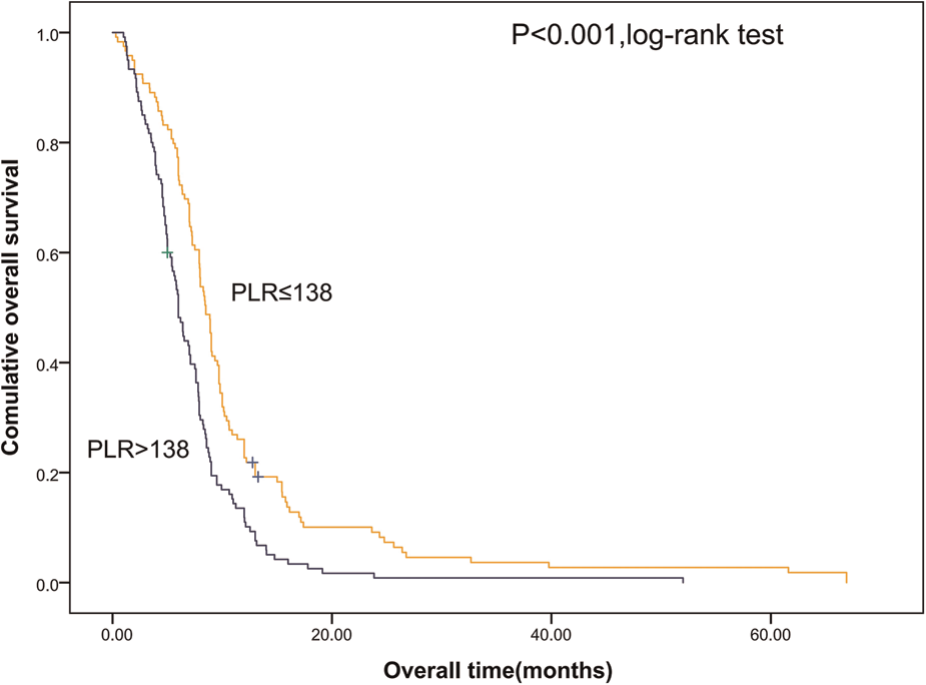
Kaplan–Meier graphs of OS for patients with high PLR (>138) and low PLR (≤138). OS, overall survival; PLR, platelet/lymphocyte count.

**Figure 3:**
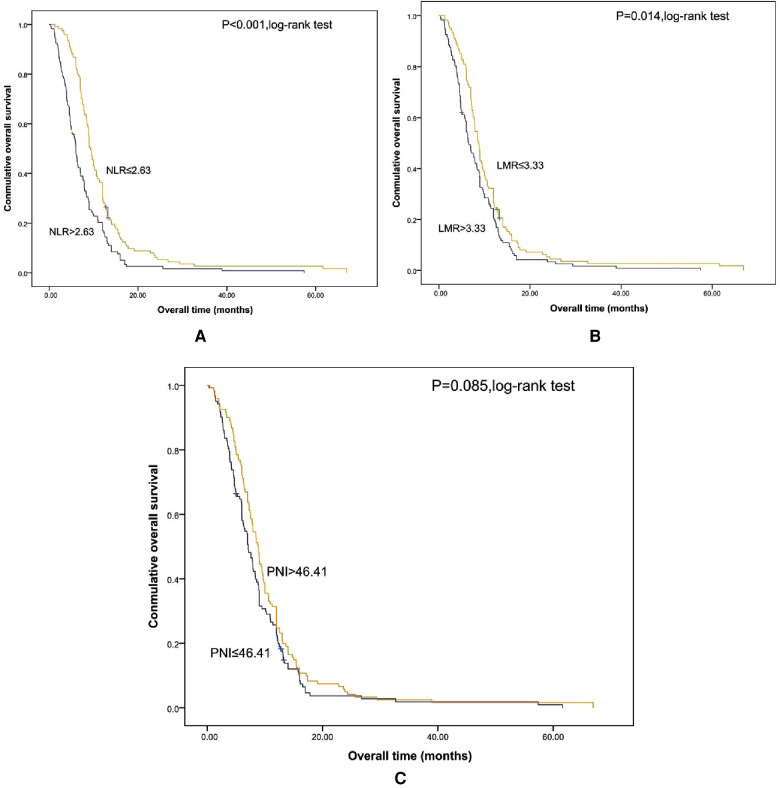
Kaplan-Meier graphs of OS for patients with NLR (**A**), LMR (**B**), and PNI (**C**). OS, overall survival; NLR, neutrophil/lymphocyte count; LMR, lymphocyte/monocyte; PNI, albumin (g/L) + 5 × lymphocyte count (10^9^/L).

Shorter OS was significantly associated with the higher SII (Figure 1). The median OS was 12.8 months and 6.56 months for patients with SII ≤ 533 and SII > 533, respectively. At the same time, we also made Kaplan–Meier curve for the relationship between the other indicators and OS. The results are shown that high PLR, NLR adn LMR were associated with short OS in the Figures 2, 3 (*p *< 0.05).

### Relationship between inflammatory markers and liver metastasis

During the collection of data, we found that some patients had liver metastases. To explore whether there was a correlation between the level of markers and early liver metastasis, we divided the patients into liver metastases (102, 42.0%) and non-liver metastases (140, 57.6%) groups and performed the chi-square test with the two groups and inflammatory markers.

As illustrated in Table 3, there were significant differences of inflammatory markers between the non-liver metastasis group and the liver metastasis group. SII, PLR and NLR in the liver metastasis group were significantly higher than that in the non-liver metastasis group, whereas PNI and LMR were lower in liver metastasis group. These results suggest that the levels of inflammatory markers were associated with early liver metastasis.

**Table 3:** Relationship between inflammation markers and liver metastasis.

	Liver metastasis	Non-liver metastasis	*p*
SII	1011.42 (1550.32)	729.86 (1060.81)	0.003
PLR	206.26 (10.67)	149.48 (82.23)	0.041
NLR	5.19 (10.67)	3.30 (3.75)	0.026
PNI	44.90 (9.14)	47.29 (7.69)	0.048
LMR	4.94 (17.79)	6.96 (29.76)	0.016

**Figure 4:**
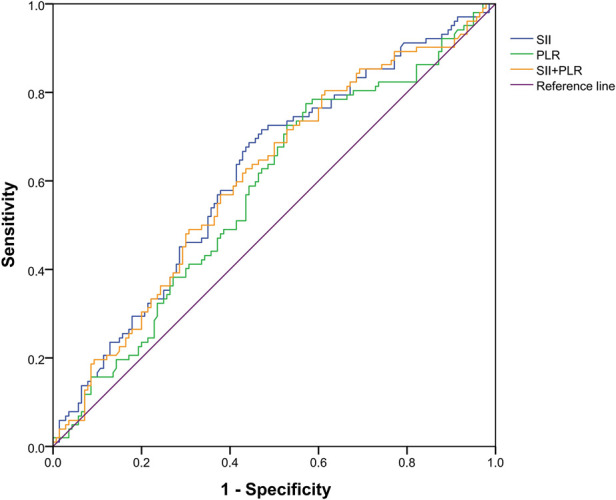
Receiver operating characteristic curve of a systemic immune inflammation index (SII), platelet-to-lymphocyte count (PLR), and SII and PLR combined. The area under the curve for SII, PLR, and both was 0.617 (*p *= 0.002), 0.575 (*p *= 0.025), and 0.606 (*p *= 0.005), respectively.

In order to verify the relationship between the combination of inflammatory markers and liver metastasis, bivariate logistic regression analyses was used to analyze the independent risk of the combination of SII and PLR, and discrimination was measured by calculating the area under the receiver operating characteristic (AUROC). As shown in Figure 4, the area under the curve for SII, PLR, and SII and PLR combined was 0.617 (*p *= 0.002), 0.575 (*p *= 0.025), and 0.606 (*p *= 0.005), respectively. These results show that SII has considerable predictive value in both liver metastasis and non-liver metastasis groups.

## Discussion

Inflammation is one of many signs of cancer. In pancreatic ductal adenocarcinoma, malignant cells are produced in the initial stage of tumorigenesis, accompanied by inflammatory cell infiltration surrounded by dense fibrosis. This inflammatory and fibrotic environment enables cancer cells to escape immune elimination and promotes malignant progression and metastasis to distant organs (22). The possibility of inhibiting or depleting tumor-promoting factors and using inflammatory cells to obtain antitumor activity in pancreatic ductal adenocarcinoma has aroused clinical and research interest (23). Inflammatory markers, especially SII, have been found related to the survival rate of a variety of cancers and the clinicopathological characteristics of tumors (24). The half-life of neutrophils is short, but their abundance in adult peripheral blood (up to 70%) suggests that they are an important cell type in the PC microenvironment (25). Tumor cells secrete proinflammatory factors, such as TNF-α and IL-12, and these factors recruit neutrophils to flow into the tumor site. In turn, neutrophils secrete numerous chemokines, such as CCL2 (MCP-1) and CCL3 (MIP-1), CCL19, and CCL20, to attract monocytes and dendritic cells into the tumor microenvironment (26). Since neutrophils ensure host survival by resolving inflammation, their role in inflammation-driven tumorigenesis is beyond doubt (27). SII has been associated with adverse outcomes in a variety of tumors, such as lung cancer, colorectal cancer, cervical cancer, and hepatocellular carcinoma (10, 11, 20, 28–31). In the present research, high SII was an independent risk marker of OS for pancreatic cancer.

New evidence indicates that platelets mediate tumor cell growth, angiogenesis, and proliferation (32, 33). Interaction of tumor cells with platelets is a prerequisite for hematogenous metastatic dissemination (34). Tumor cells can induce platelet activation and aggregation (35). PLR, as an inflammatory- and immune-based prognostic score, which can promote tumor occurrence, development, and metastasis at a high level. Results in another study (35) revealed that high PLR is a strong predictor of the prognosis of pancreatic cancer.

In the process of tumor progression, tumor cells have malignant characteristics, such as metastatic ability, resulting in an uncontrollable and life-threatening state. One of the most important events at this stage is epithelial mesenchymal transformation (EMT), through which epithelial cancer cells obtain mesenchymal characteristics, with enhanced cell movement and migration (36). EMT not only results in fibrosis after tissue injury, but also cancer progression and metastasis during wound healing. A variety of inflammatory mediators, such as TNF and IL-1 β, IL-6, IL-11 and IL-8, are reportedly effective EMT inducers (37). Remodeling of the tumor stroma is necessary for the migration and invasion of cancer cells, and inflammation participates in this process. Liver metastasis is an important factor in the clinical poor outcome of pancreatic cancer; it is one of the targets for assessing pancreatic cancer and indicates a poor prognosis. We found that the incidence of liver metastasis was higher in patients with abnormal inflammatory markers, which may be related to the promotion of tumor metastasis by inflammation.

Among the commonly used experimental indicators, carbohydrate antigen 19-9 (CA19-9) is the most commonly used biomarker for PC, in which the sensitivity and the specificity are reliable. However, a limitation of elevated levels of CA19-9 for the diagnosis of PC is that it is also increased in other routine laboratory tests such as those for biliary and gastrointestinal diseases (38). Commonly used imaging examinations, such as CT or MRI, are of great value in the diagnosis of pancreatic cancer (39). However, for patients with pancreatic cancer with no radical resection, the overall survival time is short, and the prognosis is poor. Routine CT or MRI follow-up is difficult because of poor compliance and the cost is high. Inflammatory markers are cheaper than routine examinations and may thus be used as research targets for cancer treatment in the future.

Our study has limitations. First, it had only a modest sample size of 243 patients, and it is a single-center study; thus, it lacks representativeness. Second, it is limited by its retrospective design and limited scope of analyzed outcomes. Thus, the results should be verified with larger, multi-center, prospective studies.

## Conclusions

A high SII is an independent predictor of os in patients with metastatic and unresectable PC, which is practical and easy-to-use and may help guide clinicians in the treatment of patients. The levels of SII, PLR, and NLR were higher in patients with liver metastasis, and the PNI and LMR were lower, Collectively, these findings may indicate that liver metastasis occurs readily in patients with this distribution of inflammatory markers or that PC induces an increased SII.

## Data Availability

The raw data supporting the conclusions of this article will be made available by the authors, without undue reservation.
